# Eosinophil-independent IL-5 levels are increased in critically ill COVID-19 patients who survive

**DOI:** 10.1186/s13223-023-00810-6

**Published:** 2023-07-04

**Authors:** Xiaotian Ju, Kiho Son, Rameen Jamil, Sarah Culgin, Brittany Salter, Kate Miyasaki, Nahal Emami Fard, Maria Xiao, Zil Patel, Kayla Zhang, Braeden Cowbrough, Melanie Kjarsgaard, Katherine Radford, Anna Dvorkin-Gheva, Carl D. Richards, Gerard Cox, Zain Chagla, Marek Smieja, Marcel Tunks, Waleed Alhazzani, Dawn M.E. Bowdish, Dan Perri, Parameswaran K. Nair, Roma Sehmi, Manali Mukherjee

**Affiliations:** 1 Department of Medicine, McMaster University, Hamilton, ON Canada; 2 Firestone Institute for Respiratory Health, Research Institute of St. Joe’s, Hamilton, ON Canada; 3 McMaster Immunology Research Centre, Faculty of Health Sciences, McMaster University, Hamilton, ON Canada

**Keywords:** COVID-19, Eosinophils, CD8, CD4, T cells, T2 cytokines, IL-13, IL-5, GATA-3

Dear Editor,

Eosinopenia is predictive of disease severity in COVID-19 [1], and baseline eosinophilia (in asthmatics or non-asthmatics) is associated with a lower risk of severe COVID-19 infection, usually with favourable outcomes [2]. It remains unclear whether eosinophils are directly responsible for this outcome or if they are a biomarker of a Type 2 (T2) response that skews the T1 hyperinflammatory response [1, 2]. This study investigated the airway cellular source of T2 cytokines and levels changes with worsening of COVID-19 in intubated patients.

In a prospective, single-centre cohort study, 49 mechanically ventilated patients with confirmed PCR + SARS-CoV2 infection were enrolled between October 2020 and July 2021 after providing verbal consent (patient or appropriate surrogate; Hamilton Integrated Ethics Board #10771, 11279). Blood and endotracheal aspirate (ETA) samples were collected at visit 1 (V1): time of intubation and visit 2 (V2): time of worsening based on ≥ 20% drop in lung compliance or 7 days post-intubation. All patients (demographics summarized in Table S1) were treated with 6 mg of dexamethasone daily for 10 days as per the RECOVERY trial [3]. Endotracheal aspirate (ETA) extracted cells were subject to viral inactivation with 4% cytofix prior to immunofluorescence staining and flow cytometry analyses. Cytokine storm-associated mediators (IL-1β, IL-6, IL-8, IL-10, TNFα and Oncostatin M), T2 cytokines (IL-5, IL-13) and alarmins (IL-33, soluble ST2) were measured by Ella™ multiplex or ELISA (R&D Systems, US).

Two patients were excluded for withdrawal of consent and a negative PCR at intubation, respectively. Of the 47 patients included in the final analyses, 37% had a variant of concern (VOC) and 40% had a fatal outcome. The survivor group had significantly less cardiovascular and endocrine-related comorbidities compared to the fatal population (Table S1). Blood eosinophils, comparable at V1 (0.06 ± 0.16 and 0.09 ± 0.14 ×10^9^ L) increased significantly at V2 in the survivor group only (P = 0.027) with similar increases in lymphocytes and monocytes in blood (Fig. 1A-C). Although a similar trend was seen for airway eosinophils this was not significant (Fig. 1E).

**Fig. 1:**
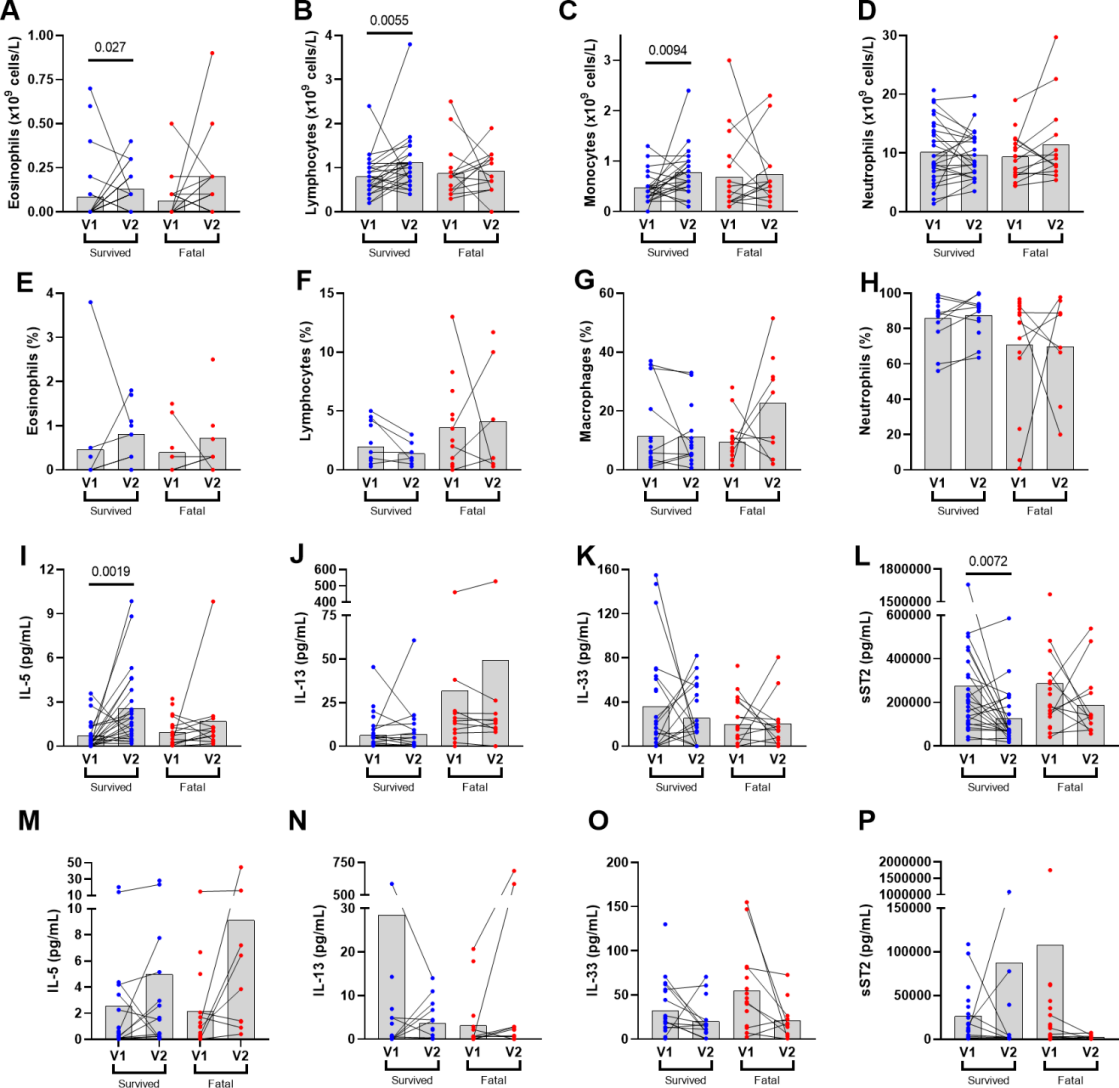
**Differential cell counts and cytokine levels in blood and endotracheal aspirates in intubated patients with severe COVID-19 infection**. Samples were collected from peripheral blood and endotracheal aspirate at time of intubation (V1) and worsening (V2) in the survival or fatal groups to assess differential cell counts (A-D and E-H, respectively) and T2 cytokines (IL-5, IL-13, IL-33, soluble ST2) (serum: I-L; ETA: M-P). Statistical analysis was done with Wilcoxon t-test (*P < 0.05 is indicative of significant difference)

Serum IL-5 increased and soluble ST2 levels decreased significantly from V1 to V2 in the survivor group (P = 0.002 and P = 0.01, respectively) (Fig. 1I-L). Although airway IL-5 levels increased from V1 to V2 in the survivor group this was not significant. However, there were significant correlations between serum IL-5 and airway IL-5 (R = 0.20, P = 0.01) and blood eosinophils (R = 0.53, P = 0.0001). As part of the cytokine storm, although serum IL-1β (P = 0.002) and ETA IL-8 (P = 0.048) increased from V1 to V2 in the survivor group (Figure S1), a significant negative correlation was found between IL-5 and IL-1β in ETA from the survivor group at V2 (R=-0.50, P = 0.018).

To investigate cell sources of T2 cytokines, ETA derived cells and blood samples were subject to flow cytometric analyses (Figure S2). There were significant increases in total CD8^+^ T cells and CD8^+^ T cells expressing IL-5/IL-13 (P = 0.03), GATA-3 (P = 0.03) and T-bet (P = 0.02) at V2 compared to V1 in the survival group only (Fig. 2). The data were similar for CD4^+^ T cells in ETA (Fig. 2) although these cell numbers were significantly lower than CD8^+^ T cells (Figure S3). The findings of a positive correlation between IL-5/IL-13^+^CD8^+^T cells and IL-5 levels (P = 0.01, R = 0.66) in ETA is suggestive that CD8 + T cells are a source of IL-5 in the airways. No differences in any lymphocyte population were found in blood likely because the site of pathology was within the airways (Figure S3).

**Fig. 2:**
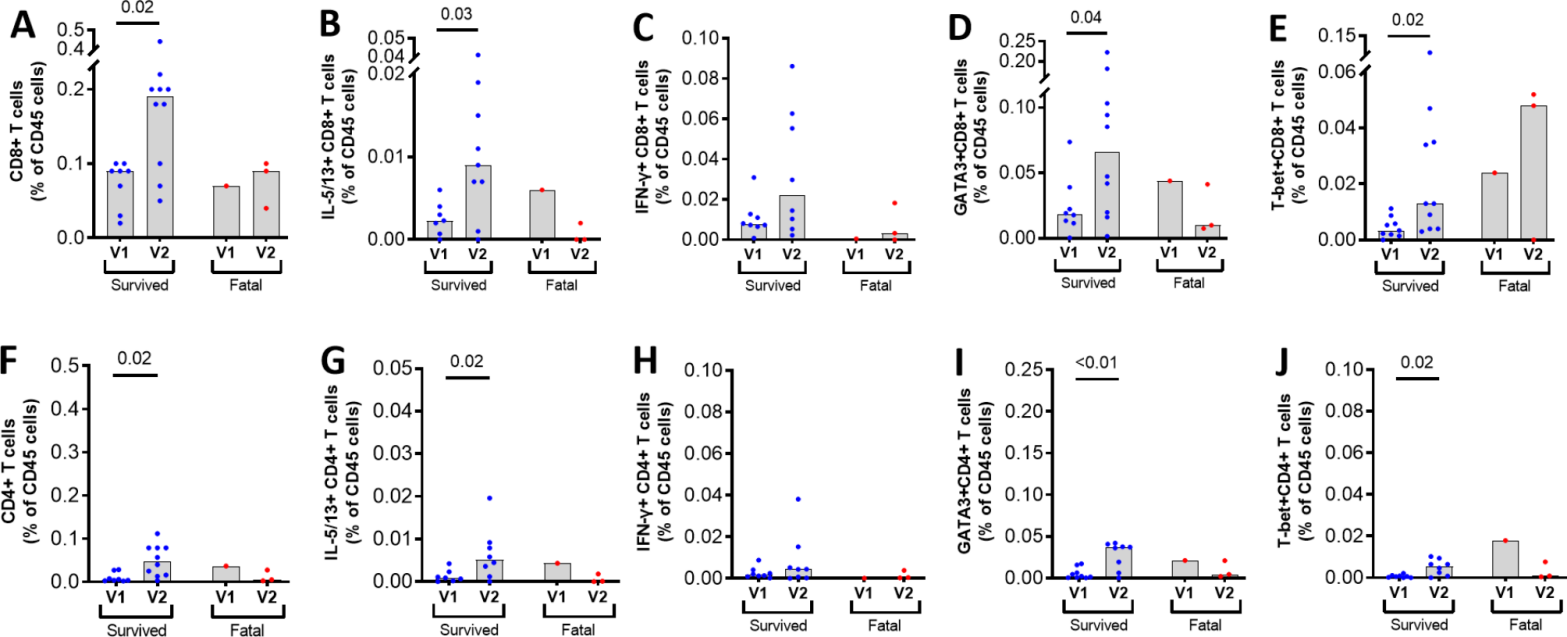
**Flow cytometric analyses of endotracheal aspirates from patients with severe COVID-19 infection**. Enumeration and phenotyping of CD8 + T cells (A-E) and CD4 + T cells (F-J) with intracellular cytokine or transcription factor expressions at time of intubation (V1) and worsening (V2) in the survival and fatal groups. Low cell recovery in ETA from the fatal group was a limiting factor in analysis. Statistical analysis for within group changes performed using Wilcoxon t-test (*P < 0.05)

This study found that IL-5, a known T2 cytokine, was detected in critically ill COVID-19 patients at time of intubation with evident increase of levels in the survivor group. The likely source of IL-5 is activated airway CD8^+^T cells which have been linked to improved survival rates in severe COVID [4, 5]. Eosinophils are unlikely to be directly contributing to favourable outcomes of critically ill COVID-19 patients but rather the activated CD8 T-cells (that are the source of IL-5 leading to eosinophilia) mediate anti-viral responses leading to improved survival.

## Electronic supplementary material

Below is the link to the electronic supplementary material.

Supplementary Material

## Data Availability

The datasets during and/or analysed during the current study available from the corresponding author on reasonable request.
