# Supplementary material for: Eosinophil-independent IL-5 levels are increased in critically ill COVID-19 patients who survive
Source: Allergy Asthma Clin Immunol. 2023 Jul 4;19:58. doi: 10.1186/s13223-023-00810-6 (PMC10318811; doi:10.1186/s13223-023-00810-6)
Supplement: Supplementary file 1 — Supplementary Material [file 13223_2023_810_MOESM1_ESM.docx]

**Online Supplement Methods:**

All patients consented to being enrolled in the study and ethics approval was obtained for this study before hand from the Hamilton Research Ethics Board (REB). REB cases are: 10771 and 11279.

**Immunofluorescence Staining and Flow Cytometric Gating Strategy**

Endotracheal aspirate (ETA)-derived cell pellets were fixed with 4% Cytofix for 45 minutes at 4^o^ C, then centrifuged and washed with PBS twice prior to immunofluorescence staining for flow cytometry. Cells were stained with antibodies to surface markers CD45-APC-H7, CD3-BV605, CD4-BV510, CD8-BV711 (BD Bioscience, Mississauga, ON, Canada). Cells were then incubated in Perm/Fix buffer (BD Bioscience, Mississauga, ON, Canada) followed by intracellular staining for transcription factors T-bet- BV421, GATA3-APC, RORγt-BV650 with relevant isotype control (BD Bioscience, Mississauga, ON, Canada). Followed by staining with intracellular cytokine staining for IFN-γ-PE-Cy7, IL-5/13-PE, IL-6-AF700 and IL-17A-BV786 with relevant isotype control (BD Bioscience, Mississauga, ON, Canada). Cells were washed and resuspended in PBS with 1% paraformaldehyde and events acquired using CytExpert software on the CytoFLEX flow cytometer (Beckman Coulter, CA, US). Data were analysed using Flow-Jo (Version 10; Tree Star, CA, US) by selecting live, singlet cells that were CD45^+^ and gating on the lympho-mononuclear region (low side scatter/low forward scatter), T lymphocytes were identified as CD3^+^CD4^+^ or CD3+CD8^+^ events. The expression of transcription factors and cytokines within CD4^+^ or CD8^+^ T cells were gated with a 98% confidence limit. The gating strategies for CD4^+^T cells and CD8^+^T cells are described in **Figure S2.** Of note, the viral inactivation with 4% Cytofix markedly affected surface receptor expression of CRTH2, which precluded the assessment of innate lymphoid cell (ILC) populations in ETA samples. In contrast, this viral inactivation strategy did not affect levels of CD45, CD3, CD4, or CD8 surface receptor expression. Data are expressed as a proportion of the gated CD45+cells. Statistical analysis was performed using Mann Whitney t-test with alpha at 0.05.

**Proteomic Assessment of Cytokines:** Multiplex assays using ELLA^TM^ platform was used to assess pro-inflammatory cytokine levels in blood serum and endotracheal aspirates.

**Table S1: Patient Clinical Characteristics**

|  | Total Patients  (N=47) | Survived Patients (N=28) | Fatal Patients  (N=19) | P Values |
| --- | --- | --- | --- | --- |
| Mean age (y [range]) | 63 (36-86) | 61 (36-83) | 65 (39-86) | 0.60 |
| Sex (Female), no (%) | 18 (38) | 10 (36) | 8 (42) | 0.66 |
| Viral Strain:  WT, no (%)  VOC, no (%) | 30 (63)  16 (34) | 17 (61)  10 (36) | 13 (68)  6 (32) | 0.70 |
| BMI (mean± SD) | 32.4 ± 11 | 34.5 ± 13 | 29.3 ± 5 | 0.36 |
| Comorbidities:  Cardiac, no (%)  Respiratory, no (%)  Hematologic, no (%)  Gastrointestinal, no (%)  Endocrine, no (%)  Renal, no (%)  Rheumatologic, no (%)  Solid Organ Malignancy, no (%)  Hematologic Malignancy, no (%) | 28 (60)  12 (26)  1 (2)  6 (13)  27 (57)  12 (26)  9 (19)  2 (4)  0 (0) | 12 (43)  6 (21)  0 (0)  4 (14)  12 (43)  6 (21)  6 (21)  1 (4)  0 (0) | 16 (84)  6 (32)  1 (5)  2 (11)  15 (79)  6 (32)  3 (16)  1 (5)  0 (0) | 0.004*  0.44  0.22  0.70  0.014*  0.44  0.63  0.78  - |
| Respiratory Illness:  Asthma, no (%)  COPD, no (%)  ILD, no (%)  Other, no (%) | 2 (4)  5 (11)  1 (1)  4 (8) | 0 (0)  4 (14)  0 (0)  3 (11) | 2 (11)  1 (5)  1 (5)  1 (5) | 0.08  0.32  0.22  0.51 |
| Comorbidity Score (mean± SD) | 2.77 ± 2 | 2.58 ± 2 | 3.0 ± 2 | 0.76 |
| APACHE II Score (mean± SD) | 20.2 ± 8 | 20.6 ± 8 | 19.5 ± 6 | 0.96 |
| Total Days Ventilated (mean± SD) | 26.4 ± 22 | 26.1 ± 23 | 27 ± 22 | 0.93 |
| Total Length of Stay (mean± SD) | 29.3 ± 22 | 28 ± 23 | 31.5 ± 22 | 0.81 |

Demographics and clinical characteristics of all study subjects.

Y: Years; WT- wild type; VOC- variants of concern; BMI- Body Mass Index; COPD – Chronic obstructive pulmonary disease; ILD – interstitial lung disease. APACHE II Score refers to score used to estimate ICU mortality.

**Figure S1: Cytokine storm levels in blood and endotracheal aspirates in intubated patients with severe COVID-19 infection.** Samples from peripheral blood (A-E) and endotracheal aspirate (F-K) taken at time of intubation (V1) and worsening (V2) in the survived or fatal groups were analysed by ELLA multiplex and ELISA assay for COVID-19 cytokine storm mediators (IL-1β, IL-6, IL-8 IL-10, TNFα and Oncostatin M (OSM), respectively. Statistical analysis was done with Wilcoxon t-test (*P<0.05 is indicative of significant difference).


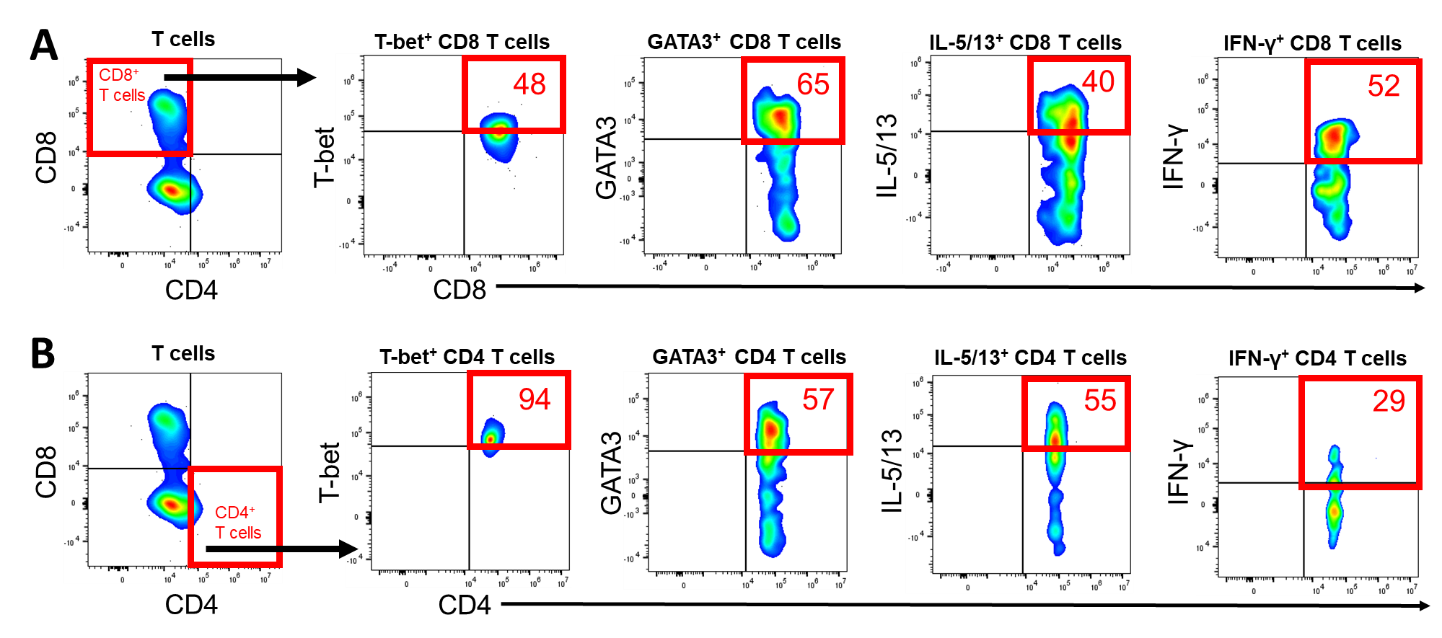


**Figure S2:** Enumeration strategy for (A) CD8+ T cells and (B) CD4+ T cells in endotracheal aspirate of patients with severe COVID-19 infection. Live, singlet cells within the lymphomononuclear region were gated as CD45+CD3+ cells and levels of co-expression of CD8 and CD4 were assessed. CD45+CD3+ CD8+ or CD4+ cells were further gated to assess transcription factor expression (T-bet, GATA-3) or cytokine levels (IL-5/13 or IFN-γ) without further stimulation.

**Figure S3:** (A) Total level of CD8+ T cells in tracheal aspirate was significantly higher than CD4+ T cells at V2 in survivor group. (B-G) There was no difference significant change in blood CD8+ or CD4+ T cell levels between visits in the any subject group.
